# GSK-3 Inhibition as a Therapeutic Approach Against SARs CoV2: Dual Benefit of Inhibiting Viral Replication While Potentiating the Immune Response

**DOI:** 10.3389/fimmu.2020.01638

**Published:** 2020-06-26

**Authors:** Christopher E. Rudd

**Affiliations:** ^1^Centre de Recherche- Maisonneuve-Rosemont Hospital (CR-HMR), Montreal, QC, Canada; ^2^Département de Medicine, Université de Montréal, Montreal, QC, Canada; ^3^Department of Medicine, Division of Endocrinology & Medical Biochemistry, McGill University Health Center, Montreal, QC, Canada

**Keywords:** T-Cells (or lymphocytes), COVID, CD8+: T lymphocyte subsets, viral (or virus), therapy

## Abstract

The SARS-CoV2 (COVID-19) pandemic and uncertainties in developing a vaccine have created an urgent need for new therapeutic approaches. A key question is whether it is possible to make rational predictions of new therapies based on the presently available scientific and medical information. In this regard, I have noticed an omission in the present analysis in the literature related to the exploitation of glycogen synthase kinase 3 (GSK-3) as a therapeutic approach. This is based on two key observations, that GSK-3 inhibitors can simultaneously block SARs viral replication, while boosting CD8+ adaptive T-cell and innate natural killer (NK) responses. Firstly, it is already clear that GSK-3 phosphorylation of SARs CoV1 N protein on key serine residues is needed for viral replication such that small molecule inhibitors (SMIs) of GSK-3 can inhibit viral replication. In comparing protein sequences, I show here that the key sites in the N protein of SARs CoV1 N for replication are conserved in SARs CoV2. This strongly suggests that GSK-3 SMIs will also inhibit SARs Cov2 replication. Secondly, we and others have previously documented that GSK-3 SMIs markedly enhance CD8+ cytolytic T-cell (CTL) and NK cell anti-viral effector functions leading to a reduction in both acute and chronic viral infections in mice. My hypothesis is that the repurposing of low-cost inhibitors of GSK-3 such as lithium will limit SARS-CoV2 infections by both reducing viral replication and potentiating the immune response against the virus. To date, there has been no mention of this dual connection between GSK-3 and SARs CoV2 in the literature. To my knowledge, no other drugs exist with the potential to simultaneously target both viral replication and immune response against SARs CoV2.

## Commentary

The SARS-CoV2 (COVID-19) pandemic has put unprecedented pressure on the economy and health of individuals around the world (1). Given the delays and potential complications in generating a vaccine, there is an urgent need for other therapeutic modalities. Possible avenues have been proposed including the use of hydroxychloroquine, anti-interleukin 7 receptor (IL7R) (Tocilizumab), inhibitors of the co-receptor serine protease TMPRSS2 (such as camostat mesylate), soluble receptor ACE2 as a decoy, amongst others (2). A key objective will be to inhibit SARs CoV2 transcription while potently boosting the immune response against the viruses and limiting the cytokine release syndrome (CRS) associated with severe disease. CRS is mediated predominately by T-cells and inflammatory myeloid lineage cells, the latter pathogenically licensed primarily by CD4+ T-cells. In this light, the potential for the use of inhibitors of the serine/threonine kinase glycogen synthase kinase 3 (GSK-3) has passed somewhat unnoticed. The hypothesis underlying this article is that GSK-3 inhibitors will both inhibit SARS-CoV2 replication and potentiate CD8+ T-cell responses for enhanced viral clearance. It should therefore be considered as a new therapeutic approach.

**Figure 1:**
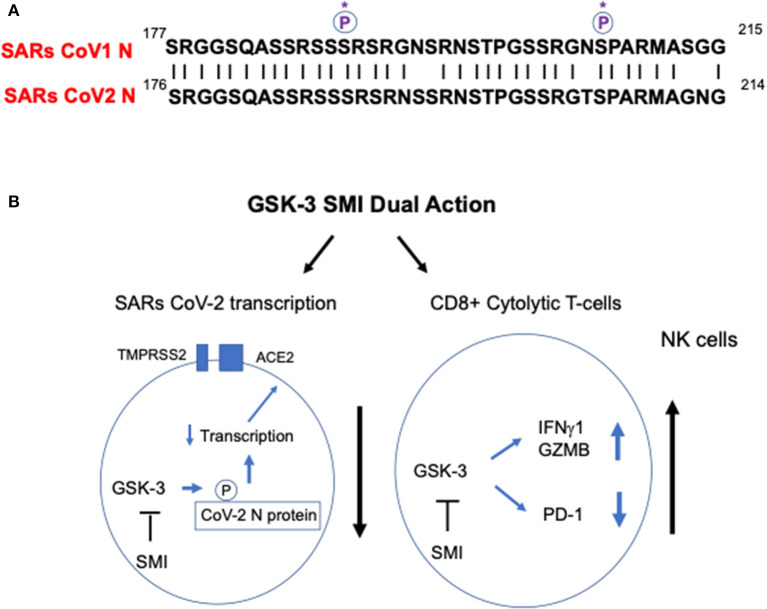
GSK-3 as a potentially important target for SARs CoV2 therapy. **(A)** A comparison of the N protein residues of SARs CoV1 and CoV2 around key serine residues 189 and 207. The critical serine phosphorylation sites on SARs CoV1 N protein needed for viral replication are conserved in SARs CoV2 (see circled P^*^). **(B)** Model for the action GSK-3 in modulating SARs CoV2 infection and the response of the T-cell immune response. GSK-3 inhibition is predicted to simultaneously inhibit SARs CoV2 N protein replication and as shown against other viruses, to preferentially boost CD8+ T-cell response against the virus. Left circle: GSK-3 inhibition is predicted to inhibit SARs CoV2 N protein phosphorylation and viral replication. Right circle: GSK-3 inhibition boosts CD8+ T-cell responses against the virus. GSK-2 blockade inhibits the expression of inhibitory receptors PD-1 and LAG3, in part, by increasing the expression of the transcription factor Tbet (*Tbx21*), while promoting the expression of cytolytic effector molecules in CD8+ T-cells such as granzyme B (GMZB) and interferon-gamma (IFNg1). GSK-2 inhibition also augments natural killer (NK) function.

The SARS-CoV2 has four structural proteins, known as the S (spike), E (envelope), M (membrane), and N (nucleocapsid) proteins. The N protein holds the RNA genome and is needed for transcription of the viral genome, while the S, E, and M proteins together create the viral envelope (3). The SARs CoV1 N protein is phosphorylated by GSK-3, an event that is needed for viral replication (4–6). The inhibition of GSK-3 with small molecule inhibitors (SMIs) prevents SARs CoV1 N1 phosphorylation, and in the process, limits SARs CoV1 viral replication (4, 5, 7). As seen in Figure 1A, in comparing sequences, it is clear that the SARs CoV2 N protein sequence has the same conserved key serine residues (serine 189 and 207) as found in the SARs CoV1 N protein. Most residues surrounding these key phosphorylation sites are identical between SARs CoV1 and SARs CoV2. It therefore seems likely that GSK-3 phosphorylation of the serine residues in SARs CoV2 will occur as in related SARs CoV1. GSK-3 inactivators also inhibit the coronovirus protease (M^pro^) (or 3C-like protease) (8) that cleaves the SARS-CoV-2 encoded polyproteins (pp1a and pp1ab) needed for viral replication and transcription (9). From these two angles, it is a reasonable hypothesis that the inhibitors of GSK-3 that inhibit SARs CoV1 replication will inhibit SARs CoV2 replication. To date, this has not been interrogated, but could be rapidly tested in *in vitro* assays using Vero6 or 293T cells.

Secondly, studies from my lab and others have shown that GSK-3 negatively regulates T-cell proliferation and function (10–12). GSK-3 is most active in resting T-cells, keeping cells in a quiescent state. This function is unlike of other kinases such as p56^lck^ which initiate the activation of T-cells (13). As a consequence, the inhibition of GSK-3 with the small molecule inhibitors (SMIs) such as SB415286 markedly enhance adaptive CD8+ cytolytic cell (CTL) function (11, 14–16) (Figure 1B). This potentiating effect is due in part to the upregulation in expression of the transcription factor T-bet (*Tbx21*) (11), a central regulator of Th1 differentiation (17). T-bet, in turn, inhibits the transcription and expression of inhibitory receptors PD-1 and LAG3, while promoting the expression of cytolytic effector molecules in CD8+ T-cells, granzyme B, perforin and interferon-gamma (16, 18). Further, and salient to this hypothesis, GSK-3 SMIs help resolve viral infections in mice, acute infection by the murine gamma-herpesvirus 68, and chronic infection with the lymphocytic choriomeningitis clone 13 (LCMV) (11). The effects of GSK-3 were preferentially seen in CD8 CTLs, and to a lesser extent, CD4+ T-cells, the latter contributing to the CRS seen in the more severe clinical manifestations of COVID-19. GSK-3 inhibitors have also been found to induce the suppressive cytokine interleukin 10 (IL-10) in CD4+ T-cells which might dampen CRS in severe disease (19). IL-10 limits the immune response and prevents tissue damage in infection and autoimmune disease (20). Lastly, GSK-3 inhibition drives the maturation and function of natural killer (NK) cells (21). Natural killer (NK) cells are effector cells of the innate immune system and also important in the control of viral infections (22). The inhibition of the GSK-3 pathway, therefore, plays central roles in promoting both the adaptive and innate immune responses against viruses.

The evidence presented here strongly suggests for the first time that GSK-3 inhibitors could constitute an effective therapy in restraining the progression of SARs CoV-2 infections. To my knowledge, no other drugs exist which might simultaneously target both SARs CoV-2 viral replication, and the immune response against the virus. It would be a novel and affordable therapeutic approach with the potential dual targeting both the virus and immune system in the treatment of COVID-19 patients. In the immediate term, lithium chloride could be administered to patients on a compassionate basis given that citrate, orotate, and carbonate salt formations of the drug are in wide clinical use for the treatment of bipolar disorders. Various side effects such as nausea have been reported, although these can be minimized by gradually increasing doses to the desired strength. Renal side effects seen in some patients can also be ameliorated with proper drug monitoring (23). Relative to the life-saving potential of GSK-3 inhibition in the treatment of COVID-19, the side effects are a minor consideration. With time, more specific GSK-3 reagents such as SB415286 could be tested in clinical trials. Both ATP competitive and potentially more selective allosteric non-competitive inhibitors could be used. The inhibitor TDZD-8 has been tested in preclinical models of cancer (24), while another inhibitor Tideglusib has been in Phase II clinical trials for Alzheimer's disease and progressive supranuclear palsy where it is well-tolerated (25, 26). Others recently noted the potential of GSK-3 inhibitors given effects on other viral infections, but failed to note the homologous regulatory phosphorylation sites in SARS CoV2 and CoV1 (27). It is these known and previously unknown target effects which I argue are key to the potential success of GSK-3 inhibition in the treatment of COVID-19. All risks should be understood prior to administration of any treatments and precautions taken under the close supervision of a physician.

## Data Availability Statement

The original contributions presented in the study are included in the article/supplementary material, further inquiries can be directed to the corresponding author/s.

## Author's Note

The SARS-CoV-2 (COVID-19) pandemic and uncertainties in developing a vaccine have created an urgent need for new therapeutic approaches. In this Opinion or Hypothesis article, I propose the exploitation of glycogen synthase kinase 3 (GSK-3) as a therapeutic approach. This is based on two key observations, that GSK-3 inhibitors can simultaneously block SARs viral replication, while boosting CD8+ adaptive T-cell and innate natural killer (NK) responses. My hypothesis is that the repurposing of low-cost inhibitors of GSK-3 such as lithium chloride will limit SARS-CoV-2 infections by both limiting viral replication and potentiating the immune response against the virus.

## Author Contributions

The author confirms being the sole contributor of this work and has approved it for publication.

## Conflict of Interest

The author declares that the research was conducted in the absence of any commercial or financial relationships that could be construed as a potential conflict of interest.
